# Genome sequence of the filamentous soil fungus *Chaetomium cochliodes* reveals abundance of genes for heme enzymes from all peroxidase and catalase superfamilies

**DOI:** 10.1186/s12864-016-3111-6

**Published:** 2016-09-29

**Authors:** Marcel Zámocký, Hakim Tafer, Katarína Chovanová, Ksenija Lopandic, Anna Kamlárová, Christian Obinger

**Affiliations:** 1 Department of Chemistry, Division of Biochemistry, University of Natural Resources and Life Sciences, Muthgasse 18, A-1190 Vienna, Austria; 2 Institute of Molecular Biology, Slovak Academy of Sciences, Dúbravská cesta 21, SK-84551 Bratislava, Slovakia; 3 Department of Biotechnology, University of Natural Resources and Life Sciences, Muthgasse 11, A-1190 Vienna, Austria

**Keywords:** *Chaetomium cochliodes*, Peroxidase-catalase superfamily, Peroxidase-cyclooxygenase superfamily, Peroxidase-chlorite dismutase superfamily, Peroxidase-peroxygenase superfamily, Heme-catalase super family

## Abstract

**Background:**

The ascomycetous family *Chaetomiaceae* (class Sordariomycetes) includes numerous soilborn, saprophytic, endophytic and pathogenic fungi which can adapt to various growth conditions and living niches by providing a broad armory of oxidative and antioxidant enzymes.

**Results:**

We release the 34.7 Mbp draft genome of *Chaetomium cochliodes* CCM F-232 consisting of 6036 contigs with an average size of 5756 bp and reconstructed its phylogeny. We show that this filamentous fungus is closely related but not identical to *Chaetomium globosum* and *Chaetomium elatum*. We screened and critically analysed this genome for open reading frames coding for essential antioxidant enzymes. It is demonstrated that the genome of *C. cochliodes* contains genes encoding putative enzymes from all four known heme peroxidase superfamilies including bifunctional catalase-peroxidase (KatG), cytochrome *c* peroxidase (CcP), manganese peroxidase, two paralogs of hybrid B peroxidases (HyBpox), cyclooxygenase, linoleate diol synthase, dye-decolorizing peroxidase (DyP) of type B and three paralogs of heme thiolate peroxidases. Both KatG and DyP-type B are shown to be introduced into ascomycetes genomes by horizontal gene transfer from various bacteria. In addition, two putative large subunit secretory and two small-subunit typical catalases are found in *C. cochliodes*. We support our genomic findings with quantitative transcription analysis of nine peroxidase & catalase genes.

**Conclusions:**

We delineate molecular phylogeny of five distinct gene superfamilies coding for essential heme oxidoreductases in *Chaetomia* and from the transcription analysis the role of this antioxidant enzymatic armory for the survival of a peculiar soil ascomycete in various harsh environments.

**Electronic supplementary material:**

The online version of this article (doi:10.1186/s12864-016-3111-6) contains supplementary material, which is available to authorized users.

## Background

The ascomycetous family of *Chaetomiaceae* (class *Sordariomycetes*) includes numerous soilborn, saprotrophic, endophytic and pathogenic fungi that apparently exhibit a large flexibility in their adaptation to various growth conditions and living niches. In Mycobank (www.mycobank.org) currently up to 451 members of this abundant fungal family are registered but only from two representatives (i.e. *Chaetomium thermophilum* and *Chaetomium globosum*) the completely sequenced genomes are available. Analysis of the genome of *C. thermophilum* [1] mainly focused on the presence of genes coding for nucleoporins of high thermal stability, whereas the draft genome of *Chaetomium globosum* [2] was mainly asked for diverse genes coding cellulolytic pathways.

The filamentous fungus *Chaetomium cochliodes* was long considered to be a variant of *Chaetomium globosum* (cf. the NCBI taxonomy database at www.ncbi.nlm.nih.gov/taxonomy) but already in very early studies e.g. [3] it was shown that *C. cochliodes* produces the antibiotic chaetomin which was shown to be highly active mainly against Gram-positive bacteria. Additionally, studies from our laboratory revealed differences between *C. globosum* and *C. cochliodes* in the primary sequence and expression profile of peroxisomal catalase-peroxidases [4]. These findings – together with the fact that peroxidases participate in diverse fungal secondary metabolism pathways [5–9] – prompted us to sequence the entire genome of *Chaetomium cochliodes* strain CCM-F232 for detailed comparative studies.

Here we release the draft genome of *C. cochliodes*, reconstruct its phylogeny and analyse the occurrence of abundant genes coding for heme containing peroxidases and catalases with respect to the recently described four distinct heme peroxidase superfamilies [10] and the heme catalase super family [11]. Interestingly, representatives from all five (super)families were found including putative bifunctional catalase-peroxidase, cytochrome *c* peroxidase, hybrid B peroxidases, cyclooxygenase-like enzymes, dye-decolorizing peroxidases, heme thiolate peroxidases as well as large- and small-subunit monofunctional catalases. The occurrence of this large number and variability of genes encoding heme hydroperoxidases in *C. cochliodes* is discussed in comparison with related fungal genomes. We support our genomic findings with a first round of a quantitative expression analysis of selected genes from all mentioned superfamilies involved in the catabolism of H_2_O_2_.

## Methods

### Source and cultivation of *Chaetomium cochliodes* and isolation of genomic DNA

*Chaetomium cochliodes* CCM F-232 was obtained from Czech Collection of Microorganisms at the Masaryk University, Faculty of Natural Sciences in Brno, Czech Republic. The composition of the incubation medium and the growth conditions were the same as described previously [4].

Genomic DNA from 100 mg of frozen fungal mycelium was isolated with the method of Carlson [12] by using 2 % CTAB in a modification suitable for genome sequencing described in [13]. Finally, extracted DNA was completely dissolved in TE buffer (10 mM Tris–HCl 1 mM EDTA, pH 8.0) to a final volume of 100 μL. The concentration of obtained sample was measured in Nanodrop 2000 (Thermo Scientific, Waltham, MA, USA).

### Library preparation for DNA sequencing

Approximately 1 μg of high quality genomic DNA was fragmented in 50 μl Low TE buffer (10 mM Tris pH 8.0, 0.1 mM EDTA) by BioRuptor UCD-200 sonication system (Life Technologies, Carlsbad, CA, USA) to obtain a population of ~190 bp long fragments. The length and the quantity of generated fragments were assessed by Bioanalyzer chip technology (Agilent Technologies, Santa Clara, CA, USA) according to the manufacturer’s instructions. The protocol of the Library Builder™ System (Life Technologies, Carlsbad, CA, USA) was used for adaptor ligation, nick repair and fragment purification. The selection of 270 bp long fragments was conducted by the Pippin Prep instrument (Sage Science, Beverly, MA, USA) according to the manufacturer’s instructions. Library quantification was carried out using the TaqMan qPCR protocol of Life Technologies.

### Genomic DNA sequencing and ORF prediction

Whole genome sequencing was carried out using the Ion Proton Technology (including the Ion AmpliSeq library preparation kit, Template OT2 200 kit, Ion PI sequencing 200 kit, and the Ion PI chip kit version 2; Life Technologies, Carlsbad, CA), according to the instructions of the manufacturer. A total of 34.746 Mbp, with a median read length of 180 bp, were assembled into a draft genome containing 6036 contigs (*N*_50_, 14,381). The genome assembly was performed with Newbler 2.9. Genome coverage of this sequencing was 316 x. The entire genome shotgun project was deposited at GenBank under accession LSBY00000000, BioProject PRJNA309375, BioSample SAMN04432217. For comparative genomic analyses of *Chaetomium cochliodes* genes Ensembl Fungi (http://fungi.ensembl.org/index.html) was used.

For gene prediction in sequenced *C. cochliodes* contigs, HMM-based methods FGENESH and FGENESH+ located at www.softberry.com [14] trained for closely related *C. globosum & C. thermophilum* were used. For all peroxidase and catalase genes they were also curated manually.

### Reconstruction of fungal phylogeny

Selected DNA sequence spanning the region from the 3′ end of the 18S rDNA, the complete ITS1, 5.8S rDNA, ITS2 and the 5′ end of the 28S rDNA from corresponding *C. cochliodes* contigs was aligned with 33 related sequences from Ascomycetes in exactly the same region obtained from GenBank (Table 1). This DNA alignment was performed with the Muscle program [15] implemented in MEGA 6 package with its default parameters and 100 iterations. For subsequent phylogeny reconstruction MEGA 6 program suite [16] was applied on this 2474 bp long DNA alignment containing the typical fungal barcode motif [17]. Maximum likelihood method with 1000 bootstrap replications and general time reversed substitution model were applied. Further, uniform rates of substitutions with invariant sites and involvement of all aligned sites with nearest-neighbour interchange and very strong branch swap filter were selected as optimised parameters. The resulting tree was rendered with Tree Explorer of the same MEGA package. For additional verification, the same 2474 bp long DNA alignment was subjected to phylogeny reconstruction using MrBayes 3.2 [18]. Majority consensus tree was obtained from all credible topologies sampled by MrBayes over 200,000 generations (with a standard deviation of split frequencies below 0.01) by using the same GTR substitution model with gamma distributed rate variation across sites and a proportion of invariable sites.

**Table 1:** List of all DNA sequences with their GenBank accession numbers used for phylogeny reconstruction in the region 18S, ITS1, 5.8S, ITS2, 28S-rDNA

Abbrev.	Fungus	Taxonom. family	GB accession #	[bp] used for phyl.
Ccoch	Chaetomium cochliodes	Chaetomiaceae	KT895345	2217
Celat	Chaetomium elatum	Chaetomiaceae	M83257	2211
Cg1	Chaetomium globosum CBS148.15	Chaetomiaceae	NT_166001	2245
Cg2	Chaetomium globosum (endophyt)	Chaetomiaceae	DQ234257	2219
Cg3	Chaetomium globosum isol. W7	Chaetomiaceae	JQ686920	2219
Cthe	Chaetomium thermophilum	Chaetomiaceae	GCA_000221225	2237
Coacu1	Colletotrichum acutatum 1	Glomerellaceae	AJ301905	2227
Coacu2	Colletotrichum acutatum 2	Glomerellaceae	AJ301906	2225
Cocir	Colletotrichum circinans	Glomerellaceae	AJ301955	2216
Cococ	Colletotrichum coccodes	Glomerellaceae	AJ301957	2218
Codem	Colletotrichum dematium	Glomerellaceae	AJ301954	2220
Colup	Colletotrichum lupini	Glomerellaceae	AJ301959	2200
Cotri	Colletotrichum trifolii	Glomerellaceae	AJ301942	2231
Cotru	Colletotricum truncatum	Glomerellaceae	AJ301937	2213
Fgram	Fusarium graminearum	Nectriaceae	NC_026477	2188
Gcin	Glomerella cingulata	Glomerellaceae	AJ301952	2198
Hgri	Humicola grisea	Chaetomiaceae	AY706334	2202
Lsak	Lecanicillium saksenae	Cordycipitaceae	AB360363	2236
Masp	Madurella sp. TMMU3956	Sordariaceae	EU815932	2271
Mhin	Myceliophthora hinnulea	Chaetomiaceae	JQ067909	2099
Mthe	Myceliophthora thermophila	Chaetomiaceae	NC_016478	2217
Mgram	Mycosphaerella graminicola	Mycosphaerellaceae	NC_018212	2195
Matr	Myrothecium atroviride	Stachybotriaceae	AJ302002	2223
Mcin1	Myrothecium cinctum 1	Stachybotriaceae	AJ301996	2204
Mcin2	Myrothecium cinctum 2	Stachybotriaceae	AJ302004	2202
Mver	Myrothecium verrucaria	Stachybotriaceae	AJ301999	2222
Ncr	Neurospora crassa	Sordariaceae	FJ360521	2230
Pan	Podospora anserina	Lasiophaeriaceae	FO904938	2196
Sfim	Sordaria fimicola	Sordariaceae	X69851	2256
Taus	Thielavia australiensis	Chaetomiaceae	JQ067908	2160
Tter	Thielavia terrestris	Chaetomiaceae	NC_016459	2233
Tasp	Trichocladium asperum	Chaetomiaceae	AY706336	2202
Tatr	Trichoderma atroviride	Hypocreacea	NW_014013638	2251
Vcil	Volutella ciliata	Nectriaceae	AJ301967	2214

### Reconstruction of molecular phylogeny of protein superfamilies

Selected protein sequences translated from *C. cochliodes* contigs (Table 2B) and similar protein sequences coding for various peroxidases and catalases (i.e. hydroperoxidases deposited at PeroxiBase http://peroxibase.toulouse.inra.fr with direct links to GenBank & UniProt) were aligned with the Muscle program [15] using default parameters and 100 iterations. Obtained alignments were inspected and ambiguously aligned regions were excluded from further analysis. Resulting alignments were subjected to protein phylogeny reconstruction using MEGA 6 [16] with optimized parameters according to lowest Bayesian information criterion scores (Additional file 1: Table S1). Maximum likelihood method with 100 bootstraps was chosen using the best substitution model for each alignment (WAG in three cases and LG in two cases cf. Additional file 1: Table S1 for details), gamma distribution of rates (four categories) and the presence of invariant sites. Nearest-neighbour interchange was used as heuristic method and very strong branch swap filter was applied. The same protein alignments were subjected to phylogeny reconstruction using MrBayes 3.2 [18]. Majority consensus tree was obtained from all credible topologies sampled by MrBayes over 500,000 generations (with a standard deviation of split frequencies below 0.10) by using the same substitution model as in MEGA. Resulting trees were rendered with FigTree graphic suite available at http://tree.bio.ed.ac.uk/software/figtree as cladograms with transformed branches.

**Table 2:** List of potentially all genes coding for enzymes involved in H2O2 metabolism in contigs of C. cochliodes genome

Gene name	In contig #	Seq. identity*	Closest neighbour**	# Introns	Gene-superfamily relations
A) genes coding for enzymes producing H2O2					
*CcochCuZnSOD*	0613	98 %	CgCuZnSOD	4	Copper/zinc superoxide dismutase superfamily (SODC)
*CcochDAAO*	0702	85 %	Mth_G2QLH3	3	Flavin D-amino acid oxidase (peroxisomal)
*CcochFeMnSOD1*	0353	91 %	TthFeMnSOD	2	Iron/manganese superoxide dismutase superfamily
*CcochFeMnSOD2*	1984 & 0805	93 %	CgFeMnSOD1	3	Iron/manganese superoxide dismutase superfamily
*CcochFeMnSOD3*	0879	94 %	CgFeMnSOD2	1	Iron/manganese superoxide dismutase superfamily
*CcochFlOx1*	0556	55 %	Colgra_E3Q5F0	1	GMC superfamily (flavin oxidases)
*CcochGlOx1*	0600	53 %	Scap_A0A084G823	7	GMC superfamily (flavin oxidases): glucose oxidase
*CcochNOx1*	0029	93 %	CgNox2	2	NADPH oxidase
B) genes coding for enzymes degrading H_2_O_2_					
*CcochkatG1*	0012	93 %	*CgkatG1*	none	peroxidase-catalase superfamily: bifunctional catalase-peroxidase
*Ccochccp*	0676	95 %	*Cgccp*	2	peroxidase-catalase superfamily: cytochrome c peroxidase
*Ccochpox2a*	3115 & 3438	68 %	*Cthepox2a*	1	peroxidase-catalase superfamily: Family II prob. manganese-dependent
*CcochhyBpox1*	3712 & 3350	100 %	*CghyBpox1*	none	peroxidase-catalase superfamily: hybrid B peroxidase
*CcochhyBpox2*	0794	93 %	*CghyBpox2*	1	peroxidase-catalase superfamily: hybrid B peroxidase
*Ccochcyox1*	2133 & 0418	83 %	*CgCyOx1*	3	peroxidase-cyclooxygenase superfamily: cyclooxygenase
*Ccochlds*	1074 & 4463	91 %	*Cglds1*	5	peroxidase-cyclooxygenase superfamily: linoleate diol synthase
*Ccochdyprx*	0391	89 %	*Cgdyprx*	none	peroxidase-dismutase superfamily: Dyp_B peroxidase (fusion w. PFL)
*Ccochhtp1*	1650	92 %	*Cghtp1*	3	peroxidase-peroxygenase superfamily: heme-thiolate peroxidase
*Ccochhtp2*	2302	95 %	*Cghtp3*	3	peroxidase-peroxygenase superfamily: heme-thiolate peroxidase
*Ccochhtp3*	1018	85 %	*Cghtp4*	2	peroxidase-peroxygenase superfamily: heme-thiolate peroxidase
*Ccochvcpo*	0469 & 1446	93 %	*Cgvcpo*	3	non heme peroxidases: vanadium haloperoxidase
*Ccochgpx*	0466	84 %	*Mthgpx*	1	non-metal peroxidases: glutathione peroxidase
*Ccoch1cysprx*	1586	96 %	*Cg1cysprx*	1	non metal peroxidases: 1-cysteine peroxiredoxin
*Ccoch2cysprx*	1595	99 %	*Cg2cysprx*	2	non-metal peroxidases: typical 2-cysteine peroxiredoxin
*CcochprxII*	0388 & 1977	95 %	*CgprxII*	1	non-metal peroxidases: atypical 2-cysteine peroxiredoxin
*CcochkatA1*	0438 & 2821	94 %	*Cgkat1*	2	heme catalase superfamily: large subunit heme catalase
*CcochkatA2*	1883 & 2899	87 %	*Cgkat2*	3	heme catalase superfamily: large subunit heme catalase
*CcochkatB1*	0511	86 %	*Cgkat3*	2	heme catalase superfamily: small subunit heme catalase
*CcochkatB2*	0351	67 %	*SschkatE*	2	heme catalase superfamily: small subunit heme catalase

* - With closest known phylogenetic neighbour

** - Abbreviations of peroxidase & catalase gene names are explained in Additional file 3: Table S2, Additional file 5: Table S4, Additional file 6: Table S3, Additional file 7: Table S5 and Additional file 8: Table S6

### Transcriptional analysis of genes involved in peroxide catabolism with RT-qPCR

To study the level of expression of genes involved in peroxide catabolism either non-induced *C. cochliodes* samples or samples induced in the early exponential phase of growth with 5 mM H_2_O_2_ or 5 mM PAA (final concentration, added only for last 30 min.) were used for total RNA isolation with RNeasy Plus Mini kit (Qiagen, Netherlands). Obtained RNA samples were directly subjected to RT-qPCR assays in AriaMx6 device (Agilent Technologies, Sancta Clara CA, USA) using the Brilliant III Ultra Fast SYBR Green Master Mix (also from Agilent Technologies) with specific primers for selected genes listed in Table 3.

**Table 3:** List of primers for *C.cochliodes* peroxidase & catalase genes

B	Primer description	Sequence in 5′ → 3′ direction	Tm [°C]	PCR prodct size [bp]
hyBpox1	CcochhyBpox1Fwd	CGAGAAAACAGATATTCTAGAAGCCA	60.1	116
	CcochhyBpox1Crev	TTCTACCGGCACCTAAATTGTT	56.5	
hyBpox2	CcochhyBpox2aFWD	GTTCATTTAGCAGGAGGTCAGG	60.3	119
	CcochhyBpox2aREV	TGTCACTGCTCGAGTTAGCATT	58.4	
cyox1	CcochCyox1bFWD	GCCTTCAAACTCCTCAACAAAG	58.4	117
	CcochCyox1bREV	GTAGCCGTCATGGAGGTTGTAT	60.3	
lds	CcochLDS3FWD	AACTTACACCATCTCCCGTGTC	58.4	127
	CcochLDS3REV	GTCGTACTGAGCGTCGTTGTAA	60.3	
dyPrx	CcochDyprxBfwd2	AAAGGAATGTCGAACCAAAAGA	54.7	135
	CcochDyprxBrev1	GCCGAGAGTAAAATCTGGAATG	58.4	
htp1	CcochHtp1fwd2	ATCTTCAACCAGACCATCTTCG	58.4	114
	CcochHtp1rev2	GAACGACTTGGACTCGATCTG	59.8	
katA2	CcochkatA2_IFWD	GAATCAACAAGACGCTTTGTGG	63.5	202
	CcochkatA2_IREV	TAGGTGGGTTAGCAAGTGAGAG	63.3	
katB1	CcochkatB2_2REV	TAAACACAAAGTCCTGGTTCCC	58.4	207
	CcochkatB1_2REV	TGGAAAAGGCGCCGTAGTCG	61.4	
katB2	CcochkatB2_1FWD	GGGGCGAGTTTGAGGTGACC	63.5	198
	CcochkatB2_2REV	TAAACACAAAGTCCTGGTTCCC	58.4	

## Results and discussion

### Overview of the sequenced genome of *Chaetomium cochliodes* CCM F-232

In total 6036 contigs were obtained from the genomic DNA of *C. cochliodes* strain CCM F-232 deposited at GenBank under accession LSBY00000000, BioProject PRJNA309375, BioSample SAMN04432217. 4141 of these contigs were larger than 500 bp. The genome size of the complete assembly was determined to be 34,745,808 bp. This value is very near to previously determined size of closely related *C. globosum* (updated to 34.9 Mb) [2]. The GC content of the entire genome of *C. cochliodes* was estimated to 55.95 % which is a slight difference to the corresponding value for *C. globosum* (55.6 %). The average size of *C. cochliodes* large genomic contigs (>500 bp) in this experiment was determined as 8256 bp, the N_50_ contig size was 14,381 bp and the largest assembled contig comprised 109,425 bp. As a quality control Phred quality scores were determined according to Illumina device: the portion of Q40^+^ bases was 34,112,976 (99.83 % of the whole genome sequence draft) whereas Q39^−^ bases portion was only 59,430 (0.17 %). Prediction of all possible ORFs of *C. cochliodes* with Chaetomia-optimised FGENESH suite [14] led in both DNA strands to a total value of 10,103. This count is lower than the estimation for mesophilic *C. globosum* [2] but much higher than the estimation for *C. thermophilum* [1] or related thermophilic fungi. A brief comparison of three related fungal genomes is presented in Table 4. The average count of exons per predicted *C. cochliodes* gene was calculated as 3 with FGENESH.

**Table 4:** comparison of three related Chaetomia genomes

Organism	Reference	Genome size [bp]	Comparison with C.coch.	Predicted ORFs
*C. cochliodes*	this work	34,745,808		10.103
*C. globosum*	[2]	34,886,900	100.41 %	11.048
*C. thermophilum*	[1]	28,322,800	81.51 %	7.165

### Phylogeny reconstruction in the 18S r DNA – ITS1 – 5.8S r DNA – ITS2 – 28S r DNA region

**Fig. 1:**
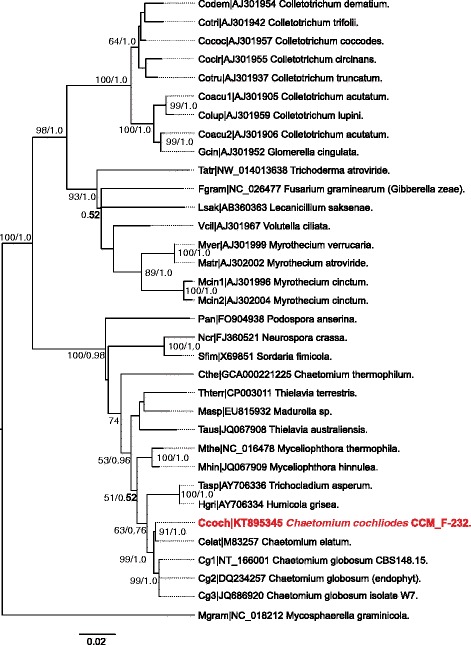
Phylogenetic relationship among 34 Ascomycetes reconstructed from the conserved region spanning 18S-ITS1-5.8S-ITS2-28S rDNA genes. Maximum likelihood method from MEGA6 with 1000 bootstraps and MrBayes method over 200,000 generations were applied on the same DNA sequence alignment 2,474 bp long (Additional file 2: Figure S1). Bootstrap values above 50 & posterior probabilities are shown, respectively. Scale bare represents the frequency of ML substitutions

First, we were interested in the exact phylogenetic position of *Chaetomium cochliodes*. For this purpose we have reconstructed the DNA phylogeny of its 2217 bp region spanning the region from the 3′ end of the 18S rDNA, the complete ITS1, 5.8S rDNA, ITS2 and the 5′ end of the 28S rDNA containing the highly conserved locus described as universal fungal barcode [17]. Besides all corresponding DNA sequences for species of the *Chaetomiaceae* family currently available in GenBank, also sequences from related ascomycetous families were included in this reconstruction (Table 1). The DNA alignment used for the phylogeny reconstruction (Additional file 2: Figure S1) reveals clear differences (i.e. substitutions, insertions and deletions) in the sequence of *C. cochliodes* if compared with corresponding sequences of *C. globosum* in the entire region. The phylogenetic output presented in Fig. 1 (obtained by two independent methods) clearly segregates *Chaetomium cochliodes* from closely related *C. elatum* which is a root-colonizing fungus whose genome is not yet sequenced [19]. Both these fungi are separated from a sister clade represented by three different DNA sequences within this region coding for various *C. globosum* strains with a high statistical support. This figure clearly demonstrates that the thermophilic representatives (mainly *C. thermophilum* but also e.g. *T. terrestris* and *M.thermophila*) of the *Chaetomiaceae* family can be considered as basal lineages of the Chaetomia clade thus suggesting that mesophily has evolved only secondarily in this lineage. Our results correlate with the previous work on thermophilic fungi [20] and particularly on the thermostability of *Chaetomiaceae* [21] where *C. cochliodes* was not included at that time.

### Putative heme peroxidases & catalases in *Chaetomium cochliodes*

Intracellular hydrogen peroxide is a by-product of various physiological pathways but, unique among all reactive oxygen species, it serves also as an important signalling molecule in apoptosis and ageing [22]. In filamentous fungi hydrogen peroxide was shown to be implicated in essential proliferation and differentiation processes [23]. Thus we have performed this genomic screening for all possible ORFs coding for a) enzymes supposed to release H_2_O_2_ during their reaction and b) two main types of enzymes involved in the catabolism of hydrogen peroxide in a novel genome of a soil Ascomycete. With TBLASTX method we could identify 8 genes for various oxidoreductases producing H_2_O_2_ (Table 2A) and up to 20 distinct genes belonging to various heme and non-heme peroxidase superfamilies as well as to the heme catalase superfamily. Overview on all these genes together with their introns composition is presented in Table 2B. All presented sequences are from contigs of the genome project deposited at GenBank under accession LSBY00000000, BioProject PRJNA309375, BioSample SAMN04432217. From Table 2 it is obvious that genes coding H_2_O_2_ degradation exhibit a higher diversity than genes coding H_2_O_2_-releasing enzymes. Detected genes for non-heme peroxidases include vanadium-containing haloperoxidase, glutathione peroxidase as well as 1-cysteine and 2-cystein peroxiredoxins. This work focuses further on genes coding for heme peroxidases.

As was presented recently, there are at least four heme peroxidase superfamilies and one heme catalase superfamily that arose independently during a convergent evolution. They differ in overall fold, active site architecture and enzymatic activities [10]. The following sections aim to discover all genes for representatives of all five superfamilies within the genome of *C. cochliodes* and to determine their exact phylogenetic positions. Heme peroxidases are found in all kingdoms of life and typically catalyse the one- and two-electron oxidation of a myriad of organic and inorganic substrates. In addition to the basal peroxidatic activity distinct families show pronounced catalase, cyclooxygenase, chlorite dismutase or peroxygenase activities.

#### Peroxidase-catalase superfamily

**Fig. 2:**
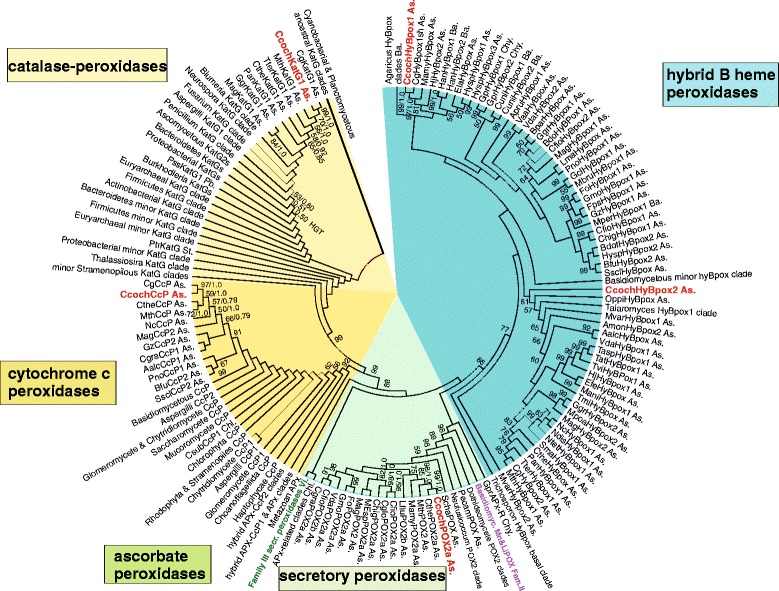
Reconstructed phylogeny of the peroxidase-catalase superfamily with focus on newly sequenced *Chaetomia* ORFs. The complete tree from 632 full length sequences with 536 sites aligned is presented with collapsed branches that do not contain any Chaetomia sequences. Distinct subfamilies are labelled in different colours. *C. cochliodes* sequences are labelled red. Values in nodes represent bootstrap values above 50 (from maximum likelihood analysis) and posterior probabilities (from Mr. Bayes), respectively. Abbreviations of peroxidase names are listed in Additional file 3: Table S2. Abbreviations of taxa: Pb, Proteobacteria; As, Ascomycota; Ba, Basidiomycota; Chy, Chytridiomycota; St, Stramenopiles; Chl, Chlorophyta; Vi, Viridiplantae

The peroxidase-catalase superfamily is currently the most abundant peroxidase superfamily in various gene and protein databases. It is comprised of three distinct families (Families I, II and III formerly known as classes) and hybrid peroxidases that represent transition forms (clades) between these families. Here we present an updated reconstruction of the phylogeny of this largest known heme peroxidase superfamily analysed previously [24, 25]. Our updated input included already 632 complete sequences and is presented in Fig. 2. We focus here on the phylogenetic positions of all representatives (ORFs) found in Chaetomia.

**Fig. 3:**
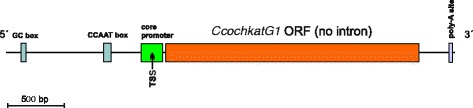
Presentation of the promoter region for *CcochkatG* gene showing typical eukaryotic regulatory elements for a HGT-related bacterial gene. Sequence analysis was performed in Contig 0012 between positions 43,000 - 47,000 with FGENESH software [14], drawn to scale

Family I of the peroxidase-catalase superfamily typically contains catalase-peroxidases (KatG), ascorbate peroxidases and cytochrome *c* peroxidases (CcP) [24]. A HGT-event from Bacteroidetes to filamentous Ascomycetes was previously reported as a peculiarity of *katG* gene family evolution [26]. Circular tree of the whole superfamily clearly demonstrates that all *katG*1 genes from the Chaetomiaceae family (cf. Additional file 3: Table S2 for abbreviations) apparently are late descendants of this HGT event (Fig. 2 left upper part). Within the upper clades we observe a basal position of the thermophilic variants from which their mesophilic counterparts descended. However, a question remains whether only the coding region of *katG*s was transferred from bacteria to fungi or whether some neighbouring regions were also included in such a transfer? We demonstrate for the gene encoding KatG1 in *C. cochliodes* (i.e. *CcochkatG*1) that the regulatory elements located on 5′ and 3′ regions embedding the ORF are clearly of eukaryotic origin (Fig. 3). In the promoter region there is (besides the GC box) a typical regulatory sequence – the “CCAAT” box involved in eukaryotic oxidative stress response [27]. In the 3′ untranslated region the poly-A site for corresponding mRNA formation can be predicted with a high probability. Thus, we can conclude that a prokaryotic *katG* was inserted in the fungal genome but received a typical eukaryotic transcription regulation during later evolution. The main physiological role of KatG in *C. cochliodes* is most propable dismutation of metabolically-generated hydrogen peroxide to molecular oxygen and water, similar to typical (monofunctional) catalases (see below) [24, 26]. In addition to KatG Chaetomia contain genes (*ccp*) encoding cytochrome *c* peroxidases (CcP, Fig. 2 – middle of the left part). The relationships among the fungi presented in the CcP phylogenetic analysis suggest that this protein has evolved vertically throughout Ascomycetes. For *ccp* genes from both *C. globosum* and *C. cochliodes* a basal lineage represented by *C. thermophilum* and *M. thermophila* is apparent in the reconstructed tree. The physiological role of CcP is still under discussion.

Further phylogenetic reconstruction of the peroxidase-catalase superfamily reveals that in *C. cochliodes* but not in *C. globosum* a Family II representative is present (Fig. 2 – lower part). This is very surprising for such closely related fungal species. However, the Family II representative from *C. cochliodes* has its closest neighbour in *C. thermophilum*. Family II ascomycetous genes code for hypothetical heme peroxidases with yet unknown reaction specificity but are closely related with well investigated basidiomycetous manganese and lignin peroxidases (Fig. 2, labelled violet). The latter are involved in oxidative degradation of lignin-containing soil debris and typically use Mn^2+^ or small organic molecules as electron donors.

Additional representatives from the peroxidase-catalase superfamily in *C. cochliodes* include two paralogs of hybrid B heme peroxidases discovered as a new gene family only recently [25]. Hybrid-type B peroxidases are present solely in fungi but are related to Family III (comprised of numerous plant secretory peroxidases, labelled green in Fig. 2) and also to Family II (fungal secretory peroxidases mentioned above). The basal lineage for the first paralog (CcochHyBpox1) together with its closely related *C. globosum* counterpart appears among mesophilic Sordariomycetes (Fig. 2 upper part). The second variant (CcochHyBpox2) containing besides the peroxidase domain also an additional C-terminal WSC (sugar binding) domain is not closely related with *C. globosum* ortholog (Fig. 2 right). Thus, both these HyBpox paralogs are not the result of a recent gene duplication but segregated rather early in the evolution of fungal genomes. Transcription analysis (Table 5 & Additional file 4: Figure S2) reveals a slight induction of both *hyBpox* genes selectively with peroxyacetic acid in the cultivation medium. In contrast, previous results [4] reveal a constitutive mode of expression for distantly related *katG1* gene with hydrogen peroxide and peroxyacetic acid.

**Table 5:** Transcription analysis of 9 selected genes for peroxide catabolism in *C. cochliodes* recorded with RT-qPCR. Quantitative values representing relative changes of the transcription level were obtained by comparison of the expression of a particular gene in 30 min. induced vs. non induced samples. The constitutively expressed ITS1 region was used as internal standard for normalization

Changes in expression levels against non-induced control*		
Analysed gene	Sample with 5 mM H_2_O_2_	Sample with 5 mM PAA
*CcochhyBpox1*	1.5 x	3.0 x
*CcochhyBpox2*	0.3 x	1.7 x
*Ccochcyox1*	0.3 x	2.3 x
*Ccochlds*	0.4 x	1.8 x
*Ccochdyprx*	3.3 x	18.5 x
*Ccochhtp1*	2.7 x	2.9 x
*CcochkatA2*	1.1 x	0.5 x
*CcochkatB1*	0.4 x	1.1 x
*CcochkatB2*	0.6 x	1.9 x

* Changes in the expression levels compared to the control sample (with the reference value of 1.0) were calculated as relative quantities due to the formula RQ = 2 ^– ΔΔCq^ where Cq is the quantification cycle of each RT-qPCR reaction. Presented are average values of triplicates for each listed gene and each inducer. Typical amplification plots and melting curves are presented in Additional file 4: Figure S2

#### Peroxidase-cyclooxygenase superfamily

Members of the peroxidase-cyclooxygenase superfamily (comprised of Families I - VII) are widely distributed among all domains of life. In many cases they are multidomain proteins with one heme peroxidase domain [10, 28]. Family IV is comprised of bifunctional cyclooxygenases possessing both peroxidase and cyclooxygenase activities. They are involved in various physiological and pathophysiological processes [29]. In mammals they are located in the luminal membrane of the endoplasmatic reticulum and mediate the conversion of free essential fatty acids to prostanoids by a two-step process [30]. The structure and function of the two distinct human paralogs (constitutive COX-1 and inducible COX-2) were intensively investigated but a comprehensive analysis of their diverse paralogs among eukaryotic microbes or even among prokaryots was only recently reported [31]. Evolutionary relationships among fungal cyclooxygenase genes were not analysed in sufficient detail yet.

**Fig. 4:**
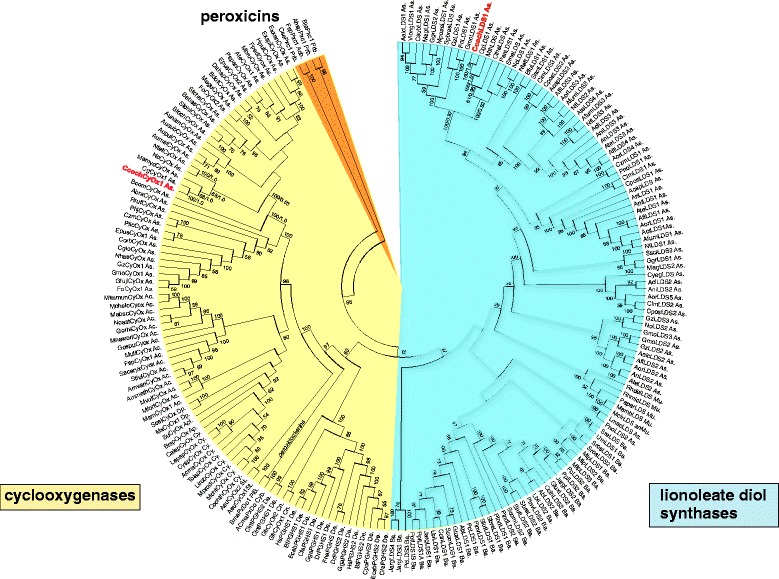
Reconstructed phylogeny of the peroxidase-cyclooxygenase superfamily with focus on *Chaetomia* ORFs. The complete tree from 204 full length sequences with 1,053 aligned sites is presented. *C. cochliodes* sequences are labelled red. Distinct subfamilies are labelled in different colours. Values in nodes represent bootstrap values above 50 (from maximum likelihood analysis) and posterior probabilities (from Mr. Bayes), respectively. Abbreviations of peroxidase names are listed in Additional file 5: Table S3. Abbreviations of taxa: Ac, Actinobacteria; Acb, Acidobacteria; Cy, Cyanobacteria; Prb, Proteobacteria; Plb, Planctomycetes (bacteria); As, Ascomycota; Ba, Basidiomycota; Mu, Mucoromycota; St, Stramenopiles; Cn, Cnidaria; De, Deuterostomia

Our current reconstruction based on the phylogeny of selected members from the whole superfamily (comprising 204 unique genes) is presented in Fig. 4. Genome analysis suggests the occurrence of two representatives of this superfamily in Chaetomia, a cyclooxygenase-like enzyme and a linoleate diol synthase. Cyclooxygenase genes from *C. cochliodes* and *C. globosum* share their closest phylogenetic neighbour (Fig. 4 upper part left) in the genome of *M. mycetomatis,* a human pathogenic fungus that grows optimally at room temperature [32]. No cyclooxygenase genes were found in thermophilic fungi so far. In contrast, the evolutionary reconstruction of another important subfamily of Family IV, linoleate diol synthases, reveals a very similar pattern for Chaetomiaceae as already described for the previous superfamily. Corresponding part of the tree (Fig. 4 – upper part right) demonstrates that genes encoding linoleate diol synthases (*lds*) from thermophilic fungi (*M. thermophila* and *C. thermophilum*) represent basal lineages for corresponding genes in mesophilic Chaetomia. Only recently it was shown that fatty acid diol synthases are unique fusion proteins containing a N-terminal heme peroxidase domain joined with a C-terminal P450-heme thiolate domain for conversion of unsaturated fatty acids to dihydroxy-fatty acids [33]. These enzymes are an essential part of the psi factor sexual inducer cascade in various fungi [34]. Their exact physiological role within the life cycle of Chaetomiaceae needs to be elucidated in the future. Our first round of transcription analysis revealed around 2-fold induction of expression of both *cyox1* and *lds* genes in a medium with peroxyacetic acid (Table 5 and Additional file 4: Figure S2).

#### Peroxidase-chlorite dismutase superfamily

**Fig. 5:**
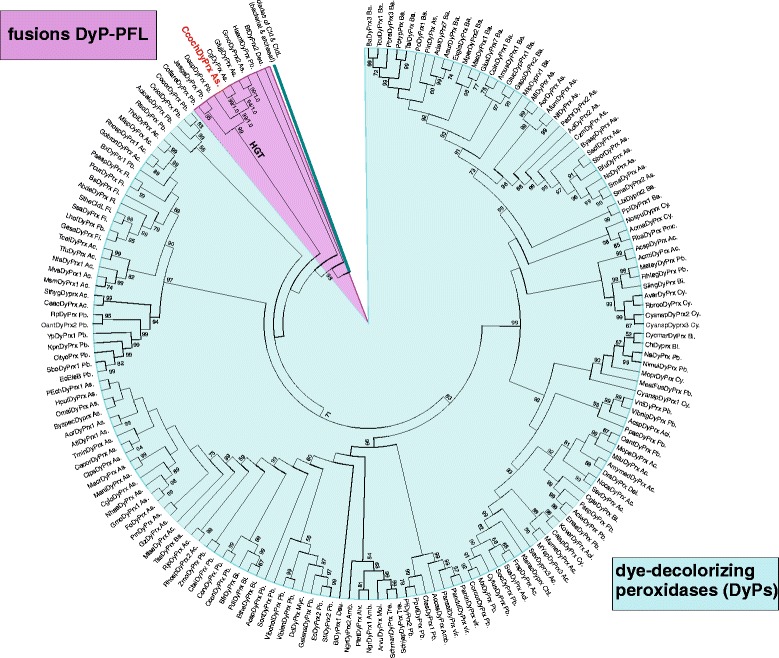
Reconstructed phylogeny of the peroxidase-dismutase superfamily with focus on newly discovered *Chaetomia* sequences forming a separate clade of DyP-Bs together with fused bacterial representatives from which they were derived by a HGT event. The complete tree from 282 full length sequences is presented with 655 sites aligned. *C. cochliodes* sequence is labelled red. Distinct subfamilies are labelled in different colours. Values in nodes represent bootstrap values above 50 (from maximum likelihood analysis) and posterior probabilities (from Mr. Bayes), respectively. Abbreviations of peroxidase names are listed in Additional file 6: Table S4. Abbreviations of taxa: vir, DNA viruses; Ac, Actinobacteria; Aci, Acidobacteria; Bi, Bacteroidetes; Chl, Chloroflexi (bacteria); Cy, Cyanobacteria; Dei, Deinococci; Fi, Firmicutes; Pb, Proteobacteria; Pmc, Planctomycetes; As, Ascomycota; Ba, Basidiomycota; Alv, Alveolata; Amb, Ameboflagellates; De, Deuterostomia; Mol, Mollusca

Our next screening within the *C. cochliodes* genome focused on the presence of genes encoding dye-decolorizing peroxidases (DyPs). These heme enzymes were first isolated from soil basidiomycetes but were further shown to be present in a wide variety of fungi and bacteria [35]. DyPs catalyse the H_2_O_2_-mediated oxidation of a very broad substrate range. Originally, fungal representatives were found to degrade bulky dyes. A detailed structure- and sequence-based comparison demonstrated that DyPs together with chlorite dismutases and chlorite-dismutase like proteins (EfeB, HemQ) constitute the CDE superfamily [36], also designated as peroxidase-chlorite dismutase superfamily [10]. The reconstructed evolution of DyPs within this superfamily is shown in Fig. 5. In fungal genomes mainly representatives of the subfamilies DyP-type D and DyP-type B can be found as paralogs. Interestingly, in the genome of *C. cochliodes* only a fused version of DyP-PFL is present, i.e. an N-terminal DyP peroxidase domain connected with a C-terminal pyruvate formate-lyase (PFL) domain known as a glycyl radical containing region [37]. This unique gene fusion was detected also in other distantly related prokaryotic & eukaryotic genomes [38]. The PFL domain can be activated by PFL activase, a radical SAM superfamily member [39], but the significance of a PFL fusion with a peroxidase domain remains elusive. We could detect a putative PFL activase in *C. cochliodes* contig 00230 revealing 81 % identity with CHGG_03160 from *C. globosum* and other putative PFL activases from filamentous fungi. Thus, *C. cochliodes* possesses both components necessary for the glycyl radical formation with yet unknown physiological function. A HGT event with a high bootstrap support in the clade of fused DyPs B can be observed between proteobacteria and ascomycetous fungi (Fig. 5 and Additional file 6: Table S4 for abbreviations). As the fused DyP B-PFL proteins are yet hypothetical, their physiological relevance has to be determined among Chaetomiaceae. Our first round of transcription analysis of *dyprx* gene exhibited the highest induction observed among all 5 superfamilies followed in this study with hydrogen peroxide (3-fold) and mainly with peroxyacetic acid (18.5-fold) in the cultivation medium (Table 5).

#### Peroxidase-peroxygenase superfamily

**Fig. 6:**
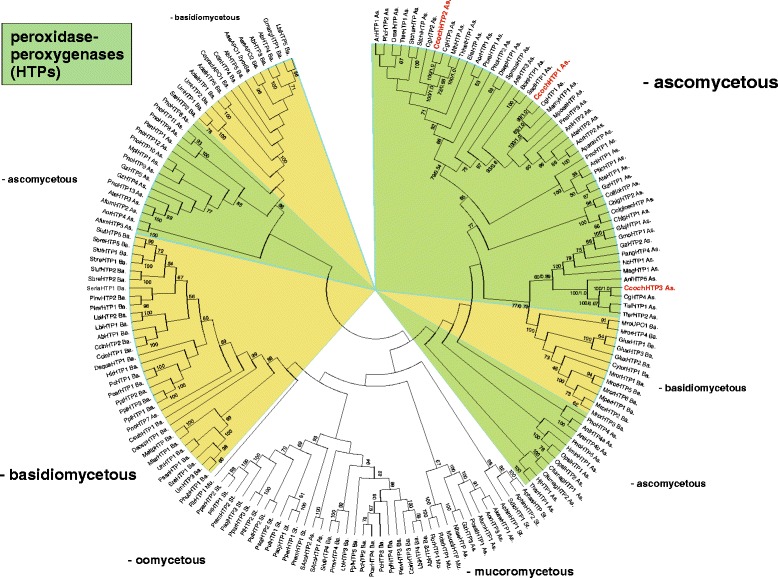
Phylogeny of the peroxidase-peroxygenase superfamily representing numerous gene paralogs of this superfamily among *Chaetomiaceae.* The complete tree from 172 full length sequences is presented with 287 sites aligned. *C. cochliodes* paralogs are labelled red. Distinct subfamilies are labelled in different colours. Values in nodes represent bootstrap values above 50 (from maximum likelihood analysis) and posterior probabilities (from Mr. Bayes), respectively. Abbreviations of peroxidase names are listed in Additional file 7: Table S5. Abbreviations of taxa: As, Ascomycota; Ba, Basidiomycota; Mu, Mucoromycota; St, Stramenopiles

Heme-thiolate peroxidases from Fungi and Stramenopiles constitute the peroxidase-peroxygenase superfamily [10]. Enzymes encoded by *htp* genes represent probably the most versatile catalysts among peroxidase superfamilies thus catalysing on one side classical heme peroxidase reactions and on the other side monooxygenase (monohydroxylation) reactions like cytochrome P450s [40]. The reconstructed phylogenetic tree for the peroxidase-peroxygenase superfamily (Fig. 6) reveals the distribution of three gene paralogs of this superfamily within the *Chaetomium cochliodes* genome. The presence of multiple gene paralogs in genomes of ascomycetous fungi is frequent and occurred by repeated gene duplications of this rather short gene but the phylogenetic distribution of *C. cochliodes* paralogs is variable (Fig. 6). Whereas there is a thermophilic basal lineage for CcochHTP2 and CcochHTP3 and their corresponding counterparts in *C. globosum*, the situation for paralog CcochHTP1 is different. Corresponding genes from pathogenic fungi represent a basal lineage for closely related CcochHTP1 and CgHTP1. It is unknown so far whether these three putative heme-thiolate peroxidases exhibit different enzymatic properties but they were segregated early during the evolution of fungal genomes and thus they all may be interesting for biotechnological applications. We have also performed transcription analysis of *htp1* gene paralog resulting in almost 3-fold induction both with hydrogen peroxide and peroxyacetic acid present in the cultivation medium (Table 5).

### Putative heme catalases in Chaetomia

**Fig. 7:**
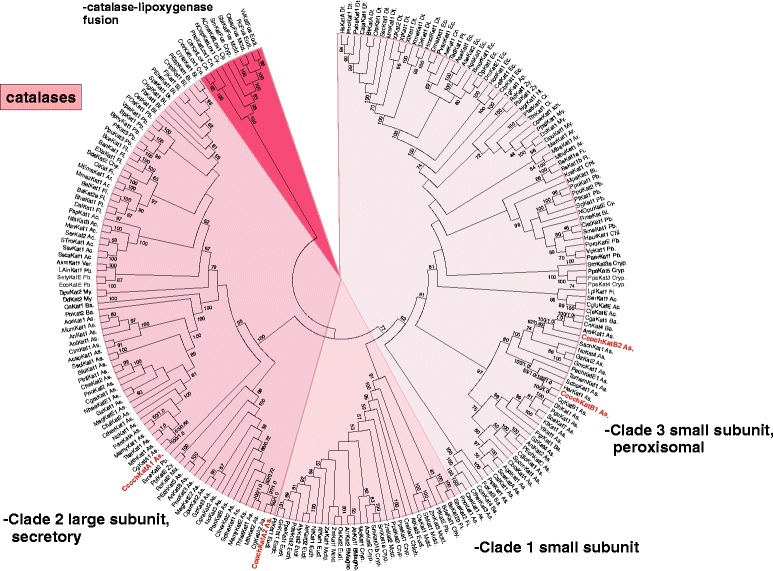
Reconstructed phylogeny of the heme catalase super family with focus on Clade 2 and 3 representing the distribution of Ascomycetous large subunit as well as small subunit catalases (labelled in different colors). The complete tree from 222 full length sequences is presented with 546 sites aligned. *C. cochliodes* paralogs are labelled red. Distinct clades are labelled in different colours. Values in nodes represent bootstrap values above 50 (from maximum likelihood analysis) and posterior probabilities (from Mr. Bayes), respectively. Abbreviations of peroxidase names are listed in Additional file 8: Table S6. Abbreviations of taxa: Ar, Archaea; Ac, Actinobacteria; Aci, Acidobacteria; Bi, Bacteroidetes; Chl, Chloroflexi (bacteria); Cy, Cyanobacteria; Dei, Deinococci; Fi, Firmicutes; Pb, Proteobacteria; Pmc, Planctomycetes; As, Ascomycota; Ba, Basidiomycota; Chy, Chytridiomycota; Zy, Zygomycota; Cn, Cnidaria; Ich, Ichthyosporea; Chlph, Chlorophyta; BMagno, basal Magnoliophyta; My, Mycetozoa; Cryp, Cryptogams, Eudi, Eudicotyledons, Mctd, Monocotyledons; De, Deuterostomia; Ec, Ecdysozoa

Typical (monofunctional) heme catalases are enzymes that very efficiently dismutase hydrogen peroxide to oxygen and water. In contrast with heme peroxidases they can both reduce and oxidize hydrogen peroxide and have negligible peroxidatic activity [41]. Heme catalases represent a monophyletic group that evolved as a distinct gene family from prokaryotes to almost all lineages of eukaryotes [11]. In Fig. 7 the phylogeny focused on fungal heme catalases is presented. There are 3 distinct clades of genes for typical catalases defined by Klotz et al. [42]. In fungi only representatives of Clade 2 (large subunit, secretory catalases) and Clade 3 (small subunit, mostly peroxisomal catalases) can be found. There are up to four gene paralogs of a catalase gene within *C. cochliodes* genome that underlines the importance of mostly monofunctional catalases for the removal of H_2_O_2_. There are thermophilic basal lineages for the large subunit secretory catalases CcochKatA1, CcochKatA2 and their *C. globosum* counterparts, a situation very similar to the peroxidase superfamilies. In contrast, there are mesophilic basal lineages for the small subunit peroxisomal catalases CcochKatB1 and CcochKatB2 (Fig. 7 – on the right). In particular, CcochKatB1 and CgKatB1 have a basal lineage among catalases from various soil and phytopathogenic fungi. Surprisingly, CcochKatB2 has no counterpart in the closely related genome of *C. globosum*. Putative catalase from a widely distributed soil fungus *S. schenckii* shares a common ancestor with this unique small subunit peroxisomal catalase of *C. cochliodes* (Fig. 7). Possible involvement of *C. cochliodes* four catalase isozymes in the defence against oxidative stress was analysed by RT-PCR. Obtained results in the early exponential phase of fungal growth show only a slight induction of the paralog *katB2* in the medium containing peroxyacetic acid (Table 5).

## Conclusions

In conlusion genomic sequence analysis revealed that *Chaetomium cochliodes* is closely related to *C. globosum & C. elatum*. These three filamentous fungi are mesophilic but probably have thermophilic ancestors as revealed from their basal lineage. *C. cochliodes* contains heme peroxidases and catalases from all so far described superfamilies. Ascomycetous genes encoding catalase-peroxidase and dye decolorizing peroxidase were obtained during the evolution by horizontal gene transfer from various bacteria. Several heme peroxidases of *Chaetomia* like hybrid heme B peroxidase, linoleate diol synthase or DyP-type B form fusions with additional functional domains that might enable a broader catalytic variability. Furthermore cytochrome *c* peroxidase, manganese and three paralogs of heme-thiolate peroxidases are found in addition to typical (monofunctional) catalases of large and small subunit architecture. Our transcription analysis reveals the highest induction of a fused *dyprx* gene with hydrogen peroxide and mainly with peroxyacetic acid in the cultivation medium followed by moderate inductions of *htp1* and *hyBpox1* genes.

## Additional files

Additional file 1:
**Table S1.** Substitution models with the lowest Bayesian information criterion scores for all 5 superfamilies analysed in this contribution. (XLSX 70 kb) Additional file 2:
**Figure S1.** DNA sequence alignment of genomic DNA from 34 ascomycetous fungi in the region covering 18S rDNA – ITS1 – 5.8S rDNA – ITS2 – 28S rDNA (fasta format). (FAS 85 kb) Additional file 3:
**Table S2.** Abbreviations of peroxidase gene names used for the peroxidase-catalase superfamily. (XLSX 54 kb) Additional file 4:
**Figure S2.** A typical profile of real-time quantitative PCR analysis of transcripts from peroxidase genes obtained from *C. cochliodes* under oxidative stress. Upper panel: amplification plots for *hyBpox*1, *cyox*1 and *lds* genes detected with SYBR Green Master Mix (Agilent Technologies). Lower panel: melting curves for *hyBpox*1, *cyox*1 and *lds* genes presented in Table 5. (TIF 450 kb) Additional file 5:
**Table S3.** Abbreviations of peroxidase gene names used for the peroxidase-cyclooxygenase superfamily. (XLSX 27 kb) Additional file 6:
**Table S4.** Abbreviations of peroxidase gene names used for the peroxidase-dismutase superfamily. (XLSX 23 kb) Additional file 7:
**Table S5.** Abbreviations of peroxidase gene names used for the peroxidase-peroxygenase superfamily. (XLSX 18 kb) Additional file 8:
**Table S6.** Abbreviations of catalase gene names used fort he catalase superfamily. (XLSX 22 kb)

## Abbreviations

CcP: Cytochrome *c* peroxidase
CldL: Chlorite dismutase-like protein
CTAB: Hexadecyltrimethylammonium bromide
HGT: Horizontal gene transfer
HMM: Hidden Markov model
KatG: Bifunctional catalase-peroxidase
LDS: Linoleate diol synthase
LiPOX: Lignin peroxidase
ML: Maximum likelihood phylogeny
MnPOX: Manganese peroxidase
ORF: Open reading frame
PAA: Peroxyacetic acid
PEG: Polyethylene glycol
PFL: Pyruvate formate-lyase
RT-qPCR: Quantitative real-time PCR
SOD: Superoxide dismutase
WSC: Cell-wall integrity & stress response component
