# Supplementary figures and images for: Genome sequence of the filamentous soil fungus Chaetomium cochliodes reveals abundance of genes for heme enzymes from all peroxidase and catalase superfamilies
Source: BMC Genomics. 2016 Sep 29;17:763. doi: 10.1186/s12864-016-3111-6 (PMC5041501; doi:10.1186/s12864-016-3111-6)

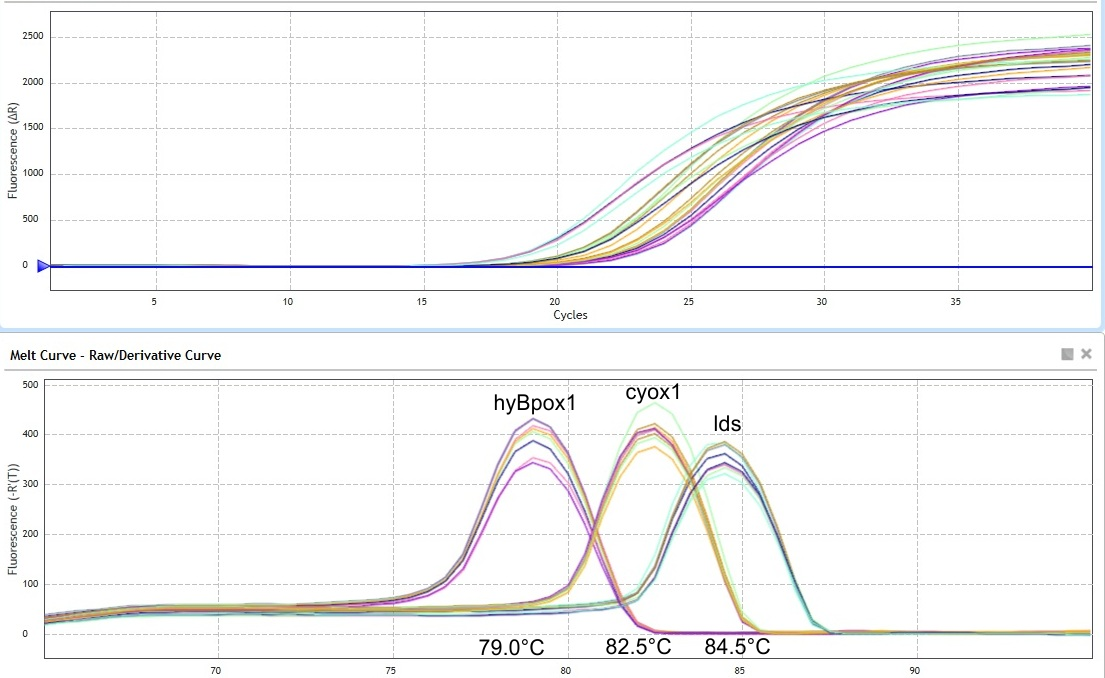

Supplement: Additional file 4: — Figure S2. A typical profile of real-time quantitative PCR analysis of transcripts from peroxidase genes obtained from C. cochliodes under oxidative stress. Upper panel: amplification plots for hyBpox1, cyox1 and lds genes detected with SYBR Green Master Mix (Agilent Technologies). Lower panel: melting curves for hyBpox1, cyox1 and lds genes presented in Table 5. (TIF 450 kb) [file 12864_2016_3111_MOESM4_ESM.tif]
